# Genetic Signatures of Dairy *Lactobacillus casei* Group

**DOI:** 10.3389/fmicb.2018.02611

**Published:** 2018-10-30

**Authors:** Alessandra Fontana, Carla Zacconi, Lorenzo Morelli

**Affiliations:** Department for Sustainable Food Process–DiSTAS, Università Cattolica del Sacro Cuore, Piacenza, Italy

**Keywords:** lactic acid bacteria, *Lactobacillus casei* group, comparative genomics, pan genome, niche adaptation

## Abstract

*Lactobacillus casei/Lactobacillus paracasei* group of species contains strains adapted to a wide range of environments, from dairy products to intestinal tract of animals and fermented vegetables. Understanding the gene acquisitions and losses that induced such different adaptations, implies a comparison between complete genomes, since evolutionary differences spread on the whole sequence. This study compared 12 complete genomes of *L. casei/paracasei* dairy-niche isolates and 7 genomes of *L. casei/paracasei* isolated from other habitats (i.e., corn silage, human intestine, sauerkraut, beef, congee). Phylogenetic tree construction and average nucleotide identity (ANI) metric showed a clustering of the two dairy *L. casei* strains ATCC393 and LC5, indicating a lower genetic relatedness in comparison to the other strains. Genomic analysis revealed a core of 313 genes shared by dairy and non-dairy Lactic Acid bacteria (LAB), within a pan-genome of 9,462 genes. Functional category analyses highlighted the evolutionary genes decay of dairy isolates, particularly considering carbohydrates and amino acids metabolisms. Specifically, dairy *L. casei/paracasei* strains lost the ability to metabolize *myo*-inositol and taurine (i.e., *iol* and *tau* gene clusters). However, gene acquisitions by dairy strains were also highlighted, mostly related to defense mechanisms and host-pathogen interactions (i.e., *yueB, esaA*, and *sle1*).

This study aimed to be a preliminary investigation on dairy and non-dairy marker genes that could be further characterized for probiotics or food applications.

## Introduction

Strains allotted to *Lactobacillus casei/Lactobacillus paracasei* group of species have been isolated from a range of ecological niches such as the different sites of the human body, breeding animals' intestinal tract and dairy environment, sourdough bread starter, and fermented vegetables, as well as from plants (Smokvina et al., 2013). A wide number of strain belonging to these two species are also commercially exploited as dairy starters, probiotics for humans and animals.

The wealth of data created by comparative genomics is allowing the identification of the core and of pan-genomes in a significant number of Lactic Acid bacteria (LAB) (Wassenaar and Lukjancenko, 2014; Sun et al., 2015; Zheng et al., 2015), allowing scientists to describe an updated evolutionary history of these bacteria and to identify genes responsible of their adaptation to an impressive number of ecological niches.

Dairy lactobacilli have been described by Stefanovic et al. (2017) as “niche specialist” (bacteria able to live in a limited number of environments) in opposition to “niche generalist” (bacteria able to live in a large number of environments) and their niche adaption have been related to their genomes enriched with sugar amino acid transportation systems as well as an enhanced proteolytic ability. In addition, dairy-adapted lactobacilli have been shown to harbor substantial gene decay, limiting their ability to survive in niche different from milk and dairy.

A relevant number of 100 *Lb. rhamnosus* genomes have been compared by Douillard et al. (2013) for their niche adaptability. They were able to cluster together most of the dairy isolates, while intestinal and probiotic strains grouped together with other human isolates. Combining phenotypic and genomic data authors were able to identify two clearly distinguishable geno-phenotypes. The “dairy group” was characterized by the absence of SpaCBA pili, lactose, maltose, and rhamnose metabolism, while the “intestinal group” harbored genes encoding for bile resistance, pili, and L-fucose utilization, traits believed to be relevant for intestinal tract survival.

On the contrary, a massive comparative genomic analysis performed by Smokvina et al. (2013) on 34 *L. paracasei* strains allowed authors to identify over 4,200 ortholog groups that comprise the pan-genome of this species, while they discover that factors generally associated with animal host-microbe interactions such as pili, cell-envelope proteinase, hydrolases p40 and p75 are part of the *L. paracasei* core genome present in all analyzed strains. What they called the *variome* consists mainly of hypothetical proteins, phages, plasmids, transposon/conjugative elements, and known functions such as sugar metabolism, cell-surface proteins, transporters. They concluded that “no specific genes or gene clusters were found to correlate with strain origin of isolation.”

A Japanese group (Toh et al., 2013) published a paper focused on the genomic adaptation of the *Lactobacillus casei*, in which genomes of *L. casei* and *L. paracasei* were compared, including in the survey also the well-known probiotic strain ATCC 53103 (LGG) belonging to the *L. rhamnosus* species. Their comparative genomic analysis identified 1,682 core genes and a wide synteny in the considered strains. However, beside this synteny, authors identified a large presence of Genomic Islands (GI) in these strains, many of which located at the genetic same loci, suggesting the presence, in the chromosomes of the *L. casei* group, of a number of hypervariable regions at the same loci. These GI could explain the adaptation to each ecological niche.

More recently, Stefanovic and McAuliffe (2018) performed a comparative genomic and metabolic analysis of three *Lactobacillus paracasei* strains, all of them isolated from the dairy environment. These authors were unable to identify a specific set of genes related to this niche, while their findings suggested a high degree of heterogeneity among strains of the species of *Lactobacillus paracasei* even if isolated from the same ecological niche.

The potential of comparative genomics as applied to the elucidation of the niche adaptation mechanisms of the *L. casei/paracasei* groups of species seems, as suggested by the above cited papers, still to be fully exploited.

The current study aimed to evaluate further genomic features of species belonging to *L. casei/paracasei* groups, to identify discriminant traits between dairy and non-dairy niche isolates. This goal was reached by focusing on complete genomes only, since evolutionary differences rely on the whole sequence and not only on gene clusters (Inglin et al., 2018). Moreover, it was shown that incomplete genomes hugely affect the core-genome identification within a species, thus high-quality input sequences are fundamental to define a reliable genomic core (Inglin et al., 2018).

Furthermore, the secretion of the lactococcal bacteriocin Lactococcin G (Moll et al., 1996) was phenotypically tested, since a dedicated ABC transporter gene (*lagD*) was identified in some of the strains investigated.

## Materials and methods

### Downloading of publicly available complete genomes and quality control

All the complete genomes of *L. casei*/*paracasei* available on the NCBI database were downloaded and the last update was done on 16 April 2018. The quality of the 19 genomes and their GC content were evaluated using Quast 4.6 (Gurevich et al., 2013), retaining high quality genomes, with N75 values >10 kbp and undetermined bases (N) per 100 kbp < 500, as suggested by Wuyts et al. (2017).

### Comparative genomic analyses

Genes were reannotated using Prokka (v1.12; Seemann, 2014) with the genus option. ANIb calculation (Richter and Rossello-Mora, 2009) was performed using pyani (v0.2.7) to evaluate distances between all *L. casei*/*paracasei* genomes.

Pan-genome analyses were carried out with Roary (v3.11.2; Page et al., 2015) by setting strict parameters: blastp identity ≥95% and gene isolates threshold ≥99% to consider a gene as conserved. The output core gene alignment obtained with Roary was used in RAxML (v8.2.4) to build a maximum-likelihood phylogenetic tree. Genomes were clustered in R (R Development Core Team, 2008) based on the gene presence–absence distance matrix calculated with the Manhattan method. The output was then visualized by using heatmap.2 from the gplots package (Warnes et al., 2016). Orthogroups (OGs) were inferred by means of OrthoFinder (v2.2.3; Emms and Kelly, 2015) and the gene content analysis was performed by mapping the orthogroups to the optimized eggNOG bacterial database with eggNOG-mapper (v1.0.3; Huerta-Cepas et al., 2016). Statistical analysis for each functional category was carried out by performing non-metric multidimensional scaling, (NMDS) on the orthogroups count profiles for dairy and non-dairy genomes, by using the metaMDS function from the vegan package in R (Oksanen et al., 2016).

### Phenotypic evaluation of lactococcin G expression in selected lactobacilli

Gene expression of the bacteriocin Lactococcin G was evaluated by testing the growth of *Lactococcus lactis* subsp. *lactis* MG1614 in presence of a *Lactobacillus paracasei* strain containing a complete *lagD* gene (*Lactobacillus paracasei* 8700:2) and a strain with a fragmented *lagD* gene (*Lactobacillus paracasei* ATCC 334). All the strains were grown at 37°C in MRS agar plates and anaerobic conditions.

## Results and discussion

**Figure 1:**
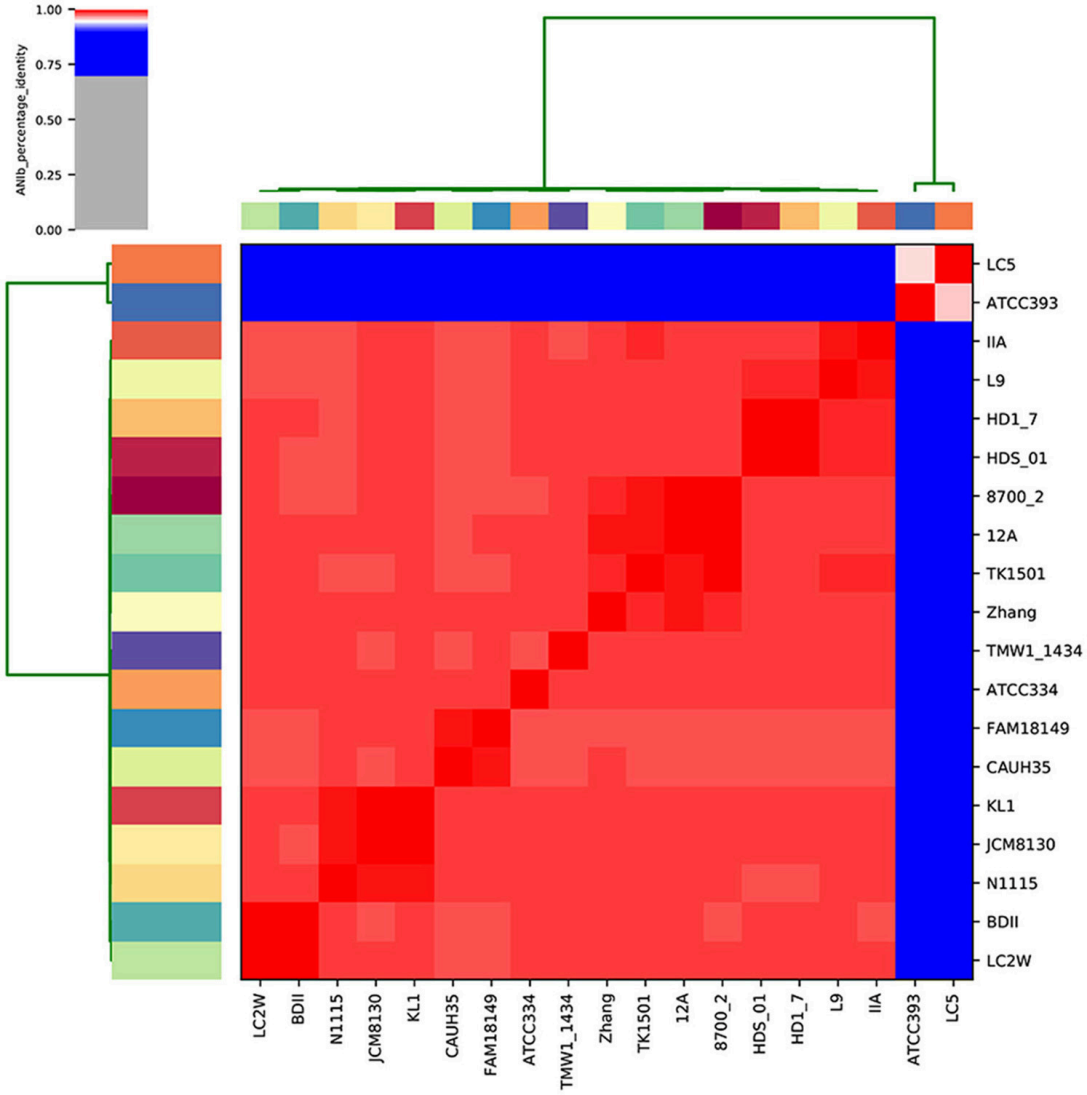
Pairwise ANIb values of the 19 genomes investigated in the study.

To evaluate what made *L. casei/paracasei* strains adapted to dairy niches (D) rather than non-dairy environments (ND), a genomic comparison was carried out on 19 publicly available complete genomes. After genomes quality checking and re-annotation, it was possible to notice that there were no appreciable differences in genomes size, GC content, and number of coding sequences (CDS) between D and ND strains (Table 1), indicating low intraspecies variation. To detect strains boundaries, pairwise genome comparisons were performed using average nucleotide identity (ANI) metrics. Particularly, ANIb distance between *L. casei*/*paracasei* strains was calculated (Figure 1) and it was evidenced the presence of two distinct clades: one represented by the dairy *L. casei* strains ATCC 393 and LC5, and the other including the remaining 17 genomes of both *L. casei*/*paracasei* strains. However, a deeper investigation of the pan-genome of such isolates is needed to better reveal the genic losses and acquisitions which led to different niches colonization.

**Table 1:** Main characteristics of the complete genomes analyzed in this study.

**Species**	**Strain**	**Origin**	**Genbank accession number**	**Genome size (Mb)**	**GC content (%)**	**No of CDS**
*L. paracasei*	ATCC 334	Dairy	GCA_000014525.1	2.92	46.58	2,854
*L. casei*	ATCC 393	Dairy	GCA_000829055.1	2.95	47.88	2,914
*L. casei*	BD-II	Dairy	GCA_000194765.1	3.13	46.29	3,091
*L. paracasei*	CAUH35	Dairy	GCA_001191565.1	2.97	46.31	2,972
*L. paracasei*	FAM18149	Dairy	GCA_002442835.1	2.97	46.31	2,989
*L. paracasei*	JCM8130	Dairy	GCA_000829035.1	3.02	46.55	2,968
*L. paracasei*	KL1	Dairy	GCA_001514415.1	2.92	46.6	2,867
*L. casei*	LC2W	Dairy	GCA_000194785.1	3.08	46.31	3,010
*L. casei*	LC5	Dairy	GCA_002192215.1	3.13	47.92	2,936
*L. paracasei*	N1115	Dairy	GCA_000582665.1	3.06	46.45	3,030
*L. paracasei*	TMW 1.1434	Dairy	GCA_002813615.1	3.17	46.37	3,009
*L. casei*	Zhang	Dairy	GCA_000019245.3	2.9	46.43	2,714
*L. casei*	12A	Corn silage	GCA_000309565.2	2.91	46.44	2,767
*L. paracasei*	TK1501	Congee	GCA_002257625.1	2.94	46.54	2,785
*L. paracasei*	HD1.7	Chinese sauerkraut	GCA_002865565.1	3.04	46.42	2,966
*L. casei*	HDS-01	Chinese sauerkraut	GCA_002902825.1	3.04	46.42	2,940
*L. paracasei*	IIA	Beef	GCA_002079285.1	3.25	46.25	3,162
*L. paracasei*	8700:2	Human intestine	GCA_000155515.2	3.02	46.3	2,928
*L. paracasei*	L9	Human intestine	GCA_001244395.1	3.08	46.35	2,962

### Core- and pan-genome analyses on complete *L. casei/paracasei* strains

**Figure 2:**
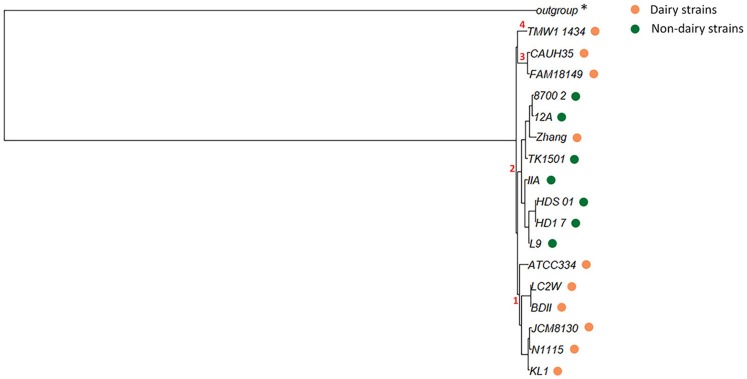
Maximum-likelihood phylogenetic tree of *L. casei*/*paracasei* strains, dairy and non-dairy niche isolates (indicated with colored dots). ^*^Outgroup represented by *L. casei* ATCC393 and *L. casei* LC5. Four clades of strains (1–4) were highlighted.

**Figure 3:**
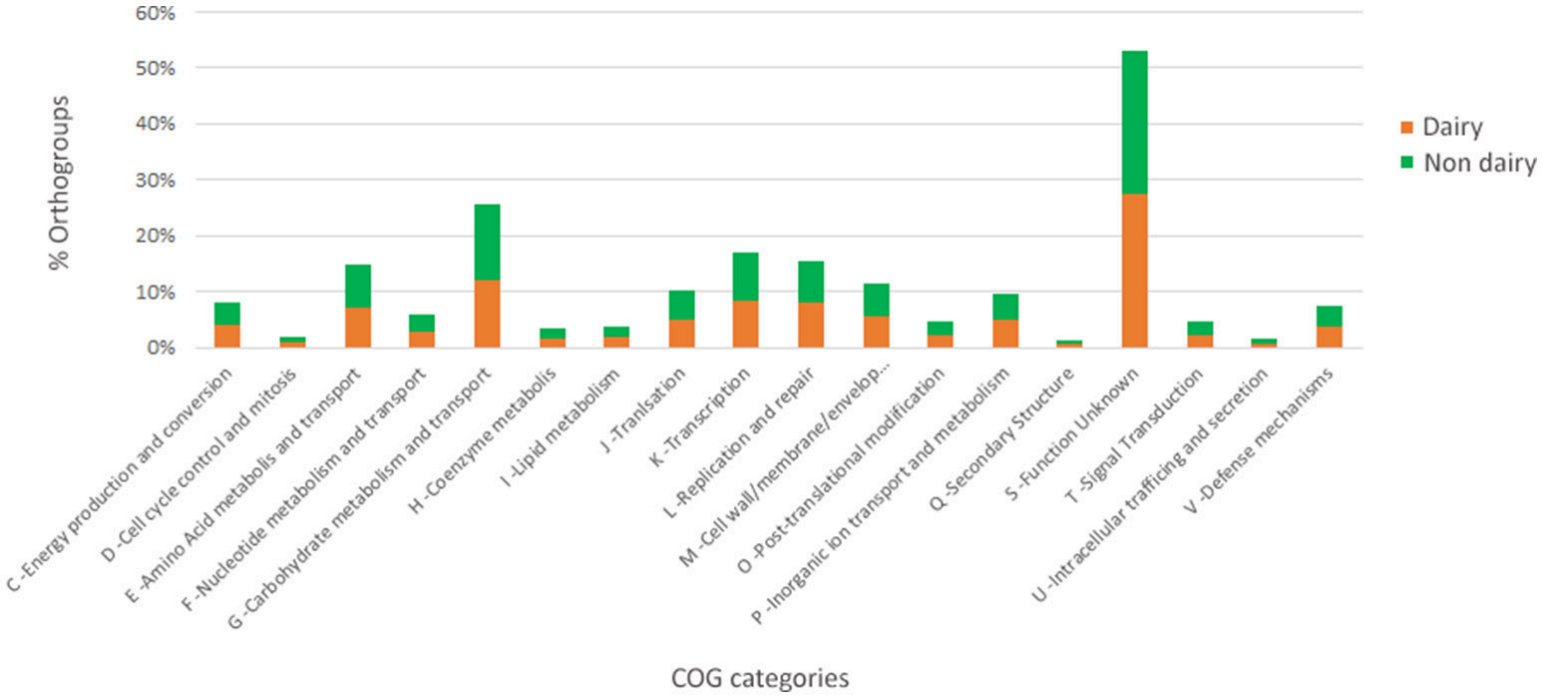
Functional COG categories related to the pan-genome of dairy and non-dairy *L. casei*/*paracasei* strains.

**Figure 4:**
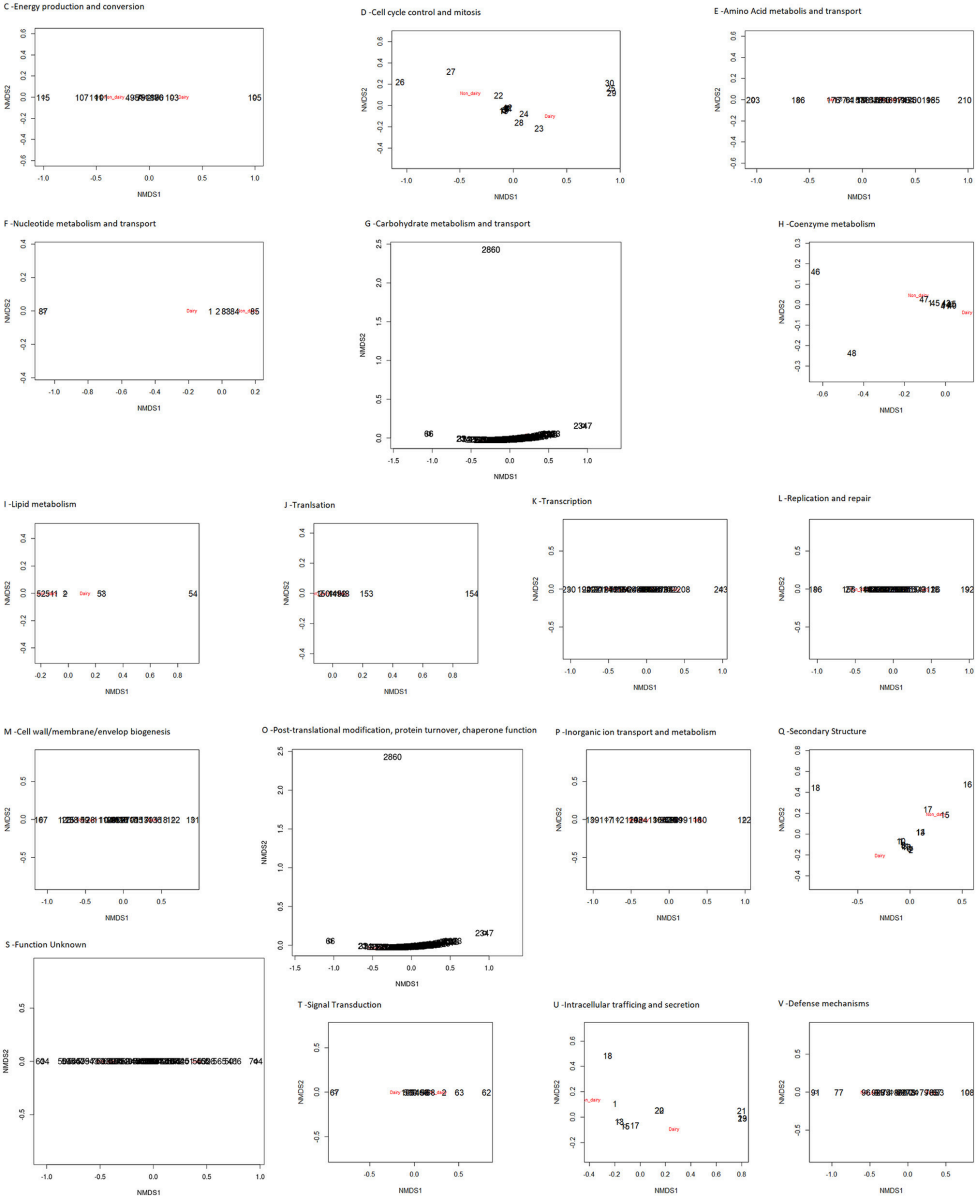
NMDS of the predicted functional capacity per niche (dairy and non-dairy), considering each COG category separately.

The presence/absence of core and accessory genes identified by Roary (Supplementary Material, Data set A) additionally revealed the lower genetic relatedness of the dairy *L. casei* strains, ATCC393 and LC5, in relation to the others. The peculiarity of these two strains, which share a high genome similarity, has been previously highlighted (Kang et al., 2017). Therefore, ATCC393 and LC5 were included in the maximum-likelihood phylogenetic tree to serve as an outgroup (Figure 2). From the inferred tree, it was possible to notice the grouping of the strains in four clades (excluding the outgroup). Specifically, ND strains were separated from D strains, except for the dairy isolate *L. casei* Zhang. Moreover, the D strain *L. paracasei* ATCC334 is represented by a single subtree separated from the other dairy niche adapted genomes. Interestingly, based on the tree inference, it is also highlighted that dairy strains originated from two distinct evolutionary events (Figure 2). The core gene alignment, on which relied the phylogenetic tree, included 313 marker genes present in 99% of the genomes investigated and with a blastp identity over 95%. Besides core genes, shell and cloud genes were also identified (3,573 and 5,576, respectively), completing a pan-genome of 9,462 genes. The 98% of the genes identified considering all genomes were clustered into 3,727 orthogroups by Orthofinder (Emms and Kelly, 2015), from which 1,791 were defined as core orthogroups (containing all species; Table 2). These orthogroups were mapped to the bacterial eggNOG database (Huerta-Cepas et al., 2016) to evaluate the main functional COG categories related to the pan-genome (Supplementary Material, Data set B). The major fraction of orthogroups (28%) were grouped under the category of “unknown function” (S). This assignment is in line with previous findings (Smokvina et al., 2013; Wuyts et al., 2017), indicating the need for further improvements in functional gene prediction. However, 13% of the OGs were grouped into the “Carbohydrate transport and metabolism” category (G), followed by “transcription” activities (K), “amino acid transport and metabolism” (E) and “inorganic ion transport and metabolism” (P; 8, 7, and 5% OGs, respectively). Interestingly, 4% of the OGs were included into the V category of “Defense mechanisms.” However, it is worth to highlight that considering the dairy and non-dairy isolates separately, the percentage of OGs grouped in the COG categories related to metabolisms was identical (Figure 3). This outcome suggests that the adaptation to the dairy environment of D strains was not reached toward metabolic simplification, but it mostly relied on a process of gene losses and acquisitions. The NMDS analysis on the orthogroups gene counts for each functional category additionally highlighted the orthogroup composition overlapping for almost all categories of dairy and non-dairy niche isolates (Figure 4). This finding is in line with a previous study (Wuyts et al., 2017), except for the U category representing the intracellular trafficking and secretion systems, which exhibited a higher separation between D and ND strains.

**Table 2:** Characteristics of the orthogroups identified by Orthofinder.

**No of genes**	**No of genes in orthogroups**	**No of unassigned genes**	**No of orthogroups**	**No of orthogroups containing all species**	**No of single-copy orthogroups**	**No of orthogroups/dairy species ±SD**	**No of orthogroups/non-dairy species ±SD**
53,868	52,813	1,055	3,727	1,791	1,652	2,712 ± 112	2,786 ± 70

**Figure 5:**
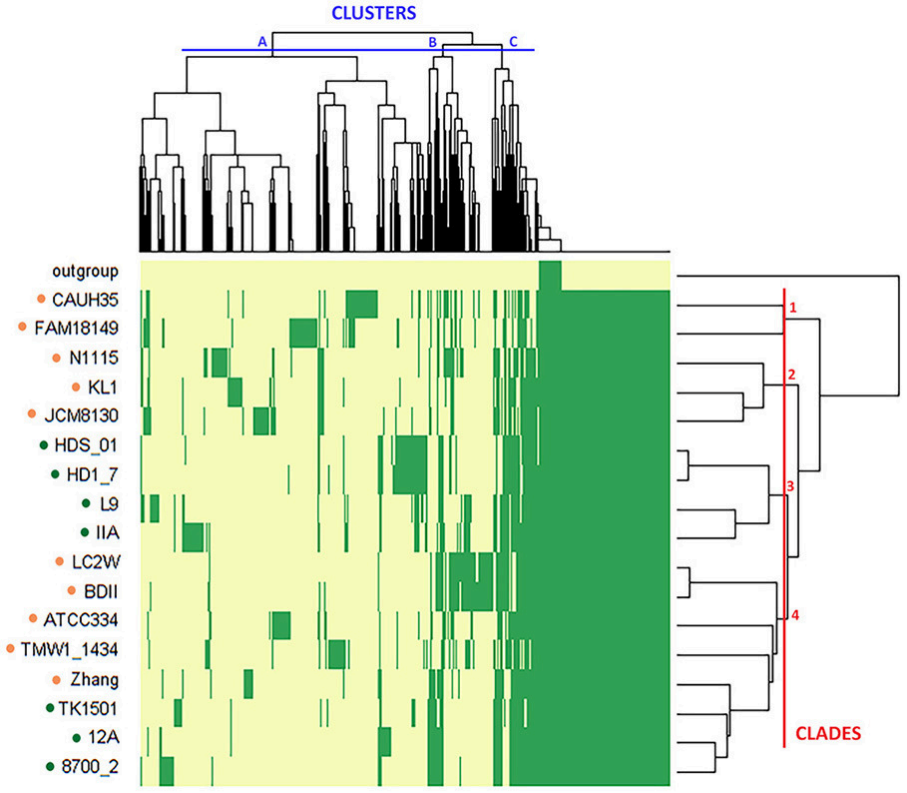
Hierarchical map and clustering of the 19 *L. casei*/*paracasei* genomes based on presence–absence of genes (yellow: absence, green: presence). The niche from which the strains were isolated is indicated by the colored dots (orange: dairy, green: non-dairy). The outgroup includes the two dairy isolated *L. casei* strains ATCC393 and LC5. OGs of genes are grouped in three clusters (A–C), while strains are grouped in four clades (1–4).

The variable genome content (i.e., variome) of the investigated strains was additionally analyzed by using the gene presence/absence matrix to build a “pan-genome” tree (Figure 5). From the hierarchical clustering it was possible to classify the OGs of genes in three clusters, whereas, at strain level, four clades were identified (excluding the outgroup). Thus, the variome-based tree recognized the same numbers of clades as the core-genome-based tree (Figure 2), but it presented a slightly different strain distribution. This outcome indicates that the discrimination between dairy and non-dairy niche adapted genomes mostly rely on the core-genome content.

Considering the KEGG categories of the pan-genome (Supplementary Material, Data set B), many genes encoded for ABC transporters and, particularly, for amino acids translocation (i.e., MetQIN for methionine, LivFGHKL for branched-chain amino acids, TcyKLMN for cystein, GlnHPQ for glutamine, ProVWX for glycin/proline, LysXY for lysine), phosphate/phosphonate (PstABCS/PhnCDE), oligopeptides (OppABCDF), cations and organic ions (i.e., manganese, zinc, alkanesulfonates), oligo and monosaccharides (galactose oligomers/maltooligosaccharides, melibiose, ribose/xylose), and for glycerol-3-phosphate (UgpABCE). Moreover, there were found 65 genes related to phosphotransferase systems (PTS), namely N-acetylgalactosamine, alpha/beta-glucoside, cellobiose, fructose, galactitol, glucitol, lactose, mannose, mannitol, sucrose, sorbose, trehalose, ascorbate, maltose, galactose, 2-O-A-mannosyl-D-glycerate-specific components. Interestingly, there were also found 12 genes encoding for penicillin-binding protein (Pbp) under the category of peptidoglycan synthesis. Among these genes there was *pbp3*, which has been suggested to be one of the targets of intestinal bile acids, by functioning as cholate resistance factor (Hattori et al., 2017). Concerning the two-component systems (TCS) category, 68 genes have been identified, among which bacitracin-related components proteins (*braERS, bceB*). TCS enable LAB responding to many change/stress conditions, as it was elucidated by Alcántara et al. for *Lactobacillus casei* BL23 (Alcántara et al., 2011).

COG and KEGG functional categories were further investigated by exploring the presence/absence of specific genes of interest in each strain (Supplementary Material, Data set B) within the two clusters of *L. casei*/*paracasei* (dairy niche isolates and other niches adapted LAB) to find putative marker genes for such clades.

### Carbohydrate transport and metabolism

All ND strains (including both *L. casei*/*paracasei* strains) exhibit a cluster of *iol* genes (*iolABCDEGJTX*) involved in the *myo*-Inositol (MI) transport and catabolic pathway (Yebra et al., 2007). As it was found in *B. subtilis*, the *iol* cluster is regulated by the catabolite control protein A (CcpA) and the corepressor phosphocarrier protein HPr (PtsH; Miwa and Fujita, 2001), which were both found in addition to the *iol* genes. These findings suggest an adaptation of certain LAB to non-dairy niches by acquiring new genes to metabolize other compounds than lactose, as it was found in *Lactobacillus casei* BL23 by Yebra et al. (2007).

Moreover, all ND strains contain the gene coding for 2-keto-3-deoxy-6-phosphogluconate aldolase (*kdgA*), which catalyze a step of the galacturonate metabolism from pectin degradation (Slováková et al., 2002). In addition, three of these strains (*L. casei* 12A, *L. paracasei* 8700:2, and TK1501) contain the whole gene cluster *kdgKAT*, indicating an adaptation to plant carbohydrates. A similar set of genes has been identified in two *L. lactis* strains isolated from beans and cress (Siezen et al., 2008), indicating an evolutionary adaptation to plant ecological niches.

The absence of two genes in four out of seven NDs were instead highlighted in comparison to D strains, which preferentially exhibited *ugl* and *hepC* genes responsible of GAGs (glycosaminoglycans) hydrolysis (i.e., chondroitin and heparin-sulfate). GAGs can interfere with cell-cell adhesion, as it was pointed out by Martín et al. working on HeLa cells and a *Lactobacillus salivarius* strain (Martín et al., 2013).

### Amino acid transport and metabolism

ND strains showed the absence of some gene clusters related to amino acid metabolism. For instance, the *yke* genes cluster (*ykeMNOP*) was mainly identified in D strains. The high fraction of proteins in dairy products (mostly caseins) could be related to the maintenance of these genes, which are putatively responsible of amino acids import and hydrolyzation. In addition, the presence of a spermidine/putrescine-binding periplasmic protein (PotD) was identified among the dairy-niche isolates (i.e., CAUH35, FAM18149, JCM8130, KL1, N1115, TMW1_1434). Putrescine is one of the most abundant biogenic amine found in dairy products, and its production was found to be strain-specific, relying on horizontal gene transfer acquisition (Linares et al., 2011).

Interestingly, ND strains contain the genes *tauA* and *tauB* encoding for two proteins (periplasmatic and ATP-binding, respectively) involved in taurine uptake under sulfate or cysteine starvation conditions (Javaux et al., 2007). The previously tested high affinity for the substrate of TauA protein (Javaux et al., 2007) can be applied in biological dosage assays of this compound.

### Defense mechanisms

ESX secretion system protein (YueB) and ESAT-6 secretion accessory factor (EsaA) were only present in D strains. These proteins belong to the so called “type VII secretion systems” or T7S system, in Gram-positive bacteria (Abdallah et al., 2007). In *B. subtilis*, the *yueB* gene has been identified as phage receptor and homologs of this protein have been also found in other species, like *S. aureus* (São-José et al., 2004, 2006). Most of the proteins belonging to the ESX/T7S system are involved in host–pathogen interactions but a further characterization of these secretion systems is still needed (Simeone et al., 2009).

Interestingly, a N-acetylmuramyl-L-alanine amidase gene (*sle1*) coding for a peptidoglycan hydrolase (PGH) was only present in D strains. This protein was found to be involved in cell separation of *Staphylococcus aureus* (Kajimura et al., 2005). However, it is known that the anti-inflammatory properties of probiotic lactobacilli often rely on their PGHs activities (Frirdich and Gaynor, 2013), resulting in enzymes of high interest.

The presence of the *pgcA* gene (phosphoglucomutase) in all ND strains but only in 4 out of 12 D strains was also highlighted. This gene is required for the synthesis of the membrane glycolipid diglucosyl-diacylglycerol (Glc_2_-DAG), whose linkage to lipoteichoic acid (LTA) seems to be a key point during *S. aureus* infection (Gründling and Schneewind, 2007). Furthermore, it is noteworthy the presence of the lactococcin A secretion protein (LcnD) in all strains but ATCC393 and LC5 (both dairy isolates). LcnD is an accessory membrane protein which enables the secretion of Lactococcin A (LcnA), a bacteriocin produced by some strains of *Lactococcus lactis* (Stoddard et al., 1992). On the contrary, the lactococcin G ABC transporter (LagD) is specifically included in all NDs strains. The presence of the putative lactococcin G transporter gene can suggest also the inclusion of the bacteriocin gene itself. This hypothesis was verified by carrying out phenotypic trials on selected *Lactobacillus* strains to evaluate its expression.

### Lactococcin G expression trial

**Figure 6:**
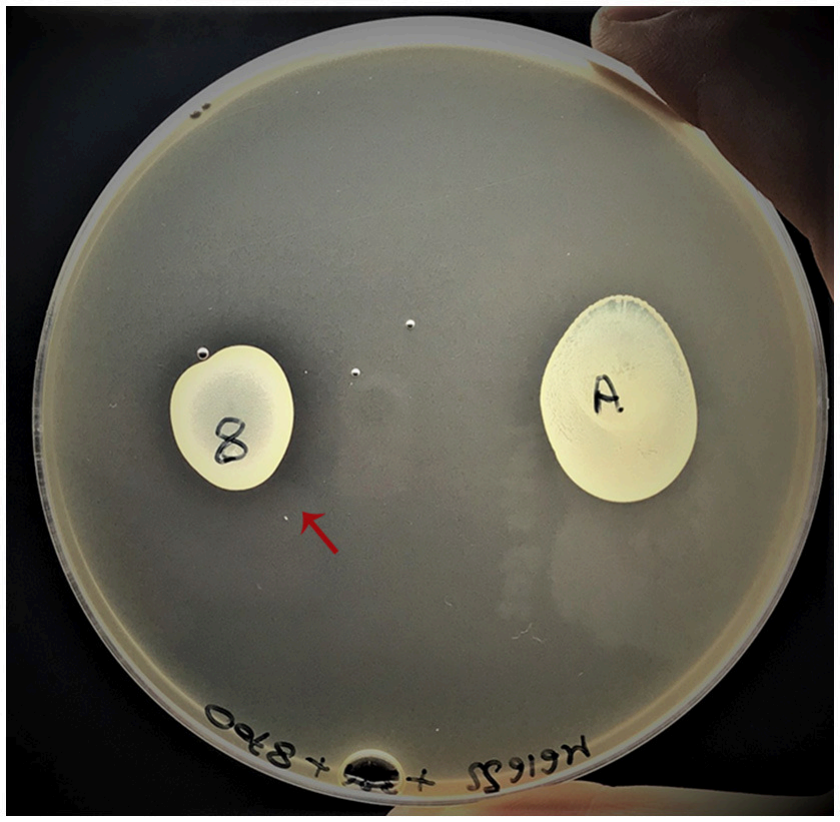
Phenotypic trial of Lactococcin G expression. The putative producer *L. paracasei* 8700:2 (indicated with the number “8”) is highlighted by a red arrow and the negative control is represented by *L. casei* ATCC334 (indicated with the letter “A”).

The secretion of Lactococcin G was tested by co-plating *L. lactis* MG1614 by inclusion and by spotting on the surface the putative bacteriocin producer *L. paracasei* 8700:2 and the non-producer (*L. casei* ATCC 334) for comparison. It was possible to appreciate an inhibition ring in proximity of the non-dairy *L. paracasei* 8700:2 (Figure 6). This data aims to be a preliminary finding for further investigations to support the expression of Lactococcin G gene among the proteins secreted by some strains of *L. casei/paracasei*.

## Conclusion

This study highlighted the origin of *L. casei/paracasei* dairy-isolated strains from two distinct evolutionary events. The variome-based analyses indicated that the discrimination between dairy and non-dairy niche adapted genomes mostly rely on the core-genome content, suggesting the presence of marker genes in dairy-niche isolated strains of *L. casei/paracasei*. These genetic signatures relied on the presence/absence of specific genes in relation to non-dairy *L. casei/paracasei* strains, supporting the occurrence of both gene gains and losses during the evolution of these LAB. Specifically, dairy-niche isolates lost many genes included in carbohydrates and amino acids metabolisms, such as the ability to utilize *myo*-inositol and taurine as carbon and sulfate sources, respectively. However, gene acquisitions have been also highlighted, especially regarding defense mechanisms. Dairy isolates indeed presented two genes (*yueB* and *esaA*) belonging to the type VII secretion systems and a peptidoglycan hydrolase (Sle1). These proteins are involved with host–pathogen interactions and further characterization should be done for probiotics development and food applications.

## Author contributions

AF performed bioinformatic analyses and wrote the manuscript. CZ supervised analyses and revised the manuscript. LM designed, supervised analyses, and revised the manuscript. All authors read and approved the final manuscript.

### Conflict of interest statement

The authors declare that the research was conducted in the absence of any commercial or financial relationships that could be construed as a potential conflict of interest.
